# Slow Recovery of Weight Bearing After Stabilization of Long-Bone Fractures Using Elastic Stable Intramedullary Nails in Children

**DOI:** 10.1097/MD.0000000000002966

**Published:** 2016-03-18

**Authors:** Patrizia Lardelli, Martina Frech-Dörfler, Stefan Holland-Cunz, Johannes Mayr

**Affiliations:** From the Department of Paediatric Surgery, University Children's Hospital Basel (Switzerland), Basel, Switzerland.

## Abstract

Stabilization of diaphyseal long-bone fractures using elastic stable intramedullary nails (ESIN) in children promises early mobilization and rapid resumption of full weight bearing.

We evaluated the duration of postoperative functional rehabilitation after ESIN, measured by the time from stabilization until first partial weight bearing, full weight bearing, and resumption of school sports.

Fifty children with unstable, displaced fractures of the femur or lower leg treated with ESIN between 2002 and 2012 were included in this retrospective analysis. We classified fractures according to the pediatric comprehensive classification of fractures (PCCF).

Thirty-five children sustained a femur fracture, and 15 children had a fracture of the lower leg or tibia. The surgeons in charge applied an additional plaster cast in 7 of 15 children who suffered a lower leg fracture. The postoperative time interval until full weight bearing in the group of children who had suffered transverse or short oblique femur fractures was significantly shorter (median: 4.4 weeks; range: 0.1–9.1 weeks) than that in the group who had sustained more complex fracture patterns (median: 6.8 weeks; range: 2.9–13.9 weeks; *P* = 0.04). Similarly, transverse and short oblique lower leg and tibia fractures required less time until full weight bearing (median: 4.1 weeks; range 2.7–6.0 weeks) than complex lower leg fractures (median: 6.1 weeks; range: 1.3–12.9 weeks; *P* = 0.04).

ESIN proved fairly effective in restoring full weight bearing in transverse or short oblique fractures of the lower extremities but was less effective in complex fractures.

## INTRODUCTION

Long-bone shaft fractures in children occur predominantly in the forearm and lower extremities.^1–3^ In the past 20 years, we observed a shift from conservative toward surgical treatment strategies due to the development of safer and more efficient surgical stabilization techniques in children. Hence, invention of the elastic stable intramedullary nailing (ESIN) technique resulted in an improvement of fracture stabilization.^4,5^ This minimally invasive method promises earlier postoperative mobilization and, consequently, earlier return to full weight bearing.^4,6^ Fast functional rehabilitation is associated with a shorter hospital stay and lower costs.^5,7^ Furthermore, this strategy, which is highly accepted by children and their caregivers, is associated with fewer long-term complications (e.g. malrotation, malalignment, limb-length discrepancies, and growth disturbances) than other stabilization modalities.^5,7–9^ Typical complications associated with ESIN are irritations of the skin caused by the protruding nail end at the nail entrance site or the occurrence of a refracture, which can interfere with the functional rehabilitation programme.^10^

The purpose of our retrospective survey was to confirm the postulated early mobilization with fast return to full weight bearing after stabilization of fractures of the femur or lower leg using ESIN.^11,12^

## PATIENTS AND METHODS

We conducted a retrospective analysis in 50 children (31 boys and 19 girls) who incurred unstable, displaced femur fractures or lower leg shaft fractures treated with ESIN between 2002 and 2012 at the University Children's Hospital Basel, Switzerland. The children were allocated to 2 groups, that is, those who had sustained a femur fracture (n = 35; group 1), and those with fractures of the lower leg or an isolated fracture of the tibia (n = 15; group 2). During the study period, 55 children were treated with ESIN. Five children (3 children with a pathological fracture, 1 child suffering from a neuromuscular disorder, and 1 child who was operated elsewhere) were excluded.

Fractures were classified according to the initial x-ray findings as transverse fractures (fracture line crossing the longitudinal axis of the injured bone at an angle of 80–90°), short oblique fractures (fracture line crossing the longitudinal axis of the injured bone at an angle of 79–60°), and long oblique or spiral fractures (fracture line crossing the longitudinal axis of the injured bone at an angle of <59°). Multifragmental fractures consisted of 3 or more fragments.

Surgical procedures had been performed as described by Ligier et al.^5^ Median follow-up period was 8.3 months (range 2.3–74.5 months). Four children were lost to follow-up.

Table 1 details the postoperative recommendations for initiation of weight bearing. Postoperative recommendation for initiation of weight bearing was at the discretion of the surgeon in charge, with no specific protocol applied. The decision was based on the level of difficulty of the reduction, fracture type, biomechanical stability of the osteosynthesis, presence of associated injuries, and the specific needs of the children and their families.

**TABLE 1:**
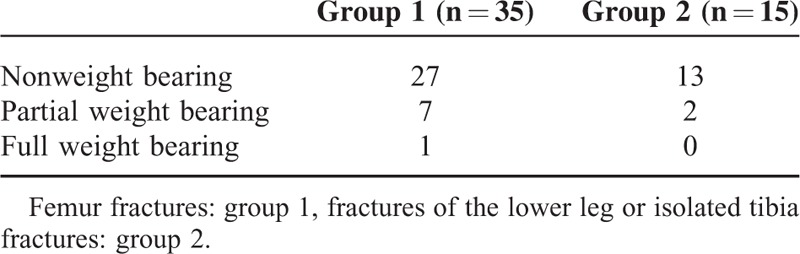
Suggested Immediate Postoperative Weight Bearing

Except for 1 child in group 1, all children were discouraged to bear weight in the immediate postoperative period. In group 2, 7 of 15 children received an additional cast in the postoperative period. Four of these children had suffered a complete fracture of the lower leg. The decision to additionally apply a cast was based on the surgeon's judgment. Duration of postoperative plaster cast immobilization was determined by the surgeon in charge, and mobilization after cast removal was started at a median of 15 days (range 2–34 days) after the operation. All children immobilized in a cast had sustained lower leg fractures.

All children underwent postoperative physiotherapy to ensure early restoration of a normal gait pattern.

The primary endpoints were the time until first partial weight bearing, time until full weight bearing, and time until resumption of school sports. Furthermore, we recorded the type of fracture based on the AO (Arbeitsgemeinschaft für Osteosynthesefragen/AO Foundation) pediatric comprehensive classification of fractures (PCCF).^13^ The fracture pattern of the fibula in fractures of the lower leg was not classified as this did not affect the type of osteosynthesis.

Associated injuries, preoperative type of immobilization or pretreatment, postoperative complications, postoperative cast immobilization, postoperative time until implant removal, residual limb-length discrepancy, residual angulation at the fracture site, shortening or malrotation of the injured long bone, need for secondary surgical intervention, functional deficits, and subjective complaints at follow-up were also documented. The data on postoperative functional outcome originated from postoperative clinical and radiological consultations carried out regularly.

### Data Analysis

We retrieved data from the patients’ medical and physiotherapeutic records and evaluated radiographs using a standardized questionnaire. We entered the validated data into a database using MS Office Excel 2010 (Microsoft Corp. Redmond, USA). Graphs and statistical analyses were generated using GraphPad Prism 5 (GraphPad Software Inc, San Diego, CA). We analyzed the data descriptively and reported median and range. The Wilcoxon test and Mann–Whitney *U* test were used, and a *P* value <0.05 was considered statistically significant.

### Ethical Standards

The local ethics committee approved the study protocol (Protocol no: 74/13) before study initiation.

## RESULTS

Overall, 35 children had sustained unstable, displaced femur shaft fractures. Nine children had suffered a fracture of the lower leg, and 6 children had an isolated fracture of the tibia. In total, 22 of 50 children had experienced a fall from height, 15 children were involved in a road traffic accident as pedestrians or cyclists, 8 children had sustained a ski/sledge injury, and 5 children had another cause of injury. Overall, median age at the time of injury was 8.6 years (range 2.3–15.7 years). The patients in group 1 had a median age of 6.5 years (range 2.3–15.7 years) at the time of surgery, whereas the median age of the patients in group 2 was 10.0 years (range 5.7–14.8 years). In both groups, there were more boys than girls; group 1 consisted of 21 boys and 14 girls, and group 2 contained 10 boys and 5 girls.

The fracture side was similarly distributed, with 16 of 35 fractures of the femur in the left side and 19 fractures on the right side. Similarly, 8 of 15 lower leg fractures were on the left side and 7 were on the right side.

The most frequently observed cause of femur fracture was a fall from height, whereas in lower leg fractures it was a road traffic injury. Table 2 shows the distribution pattern of fractures. Fifteen children (group 1) and 7 children (group 2) had more complex fracture patterns (D/5.2 II to IV).

**TABLE 2:**
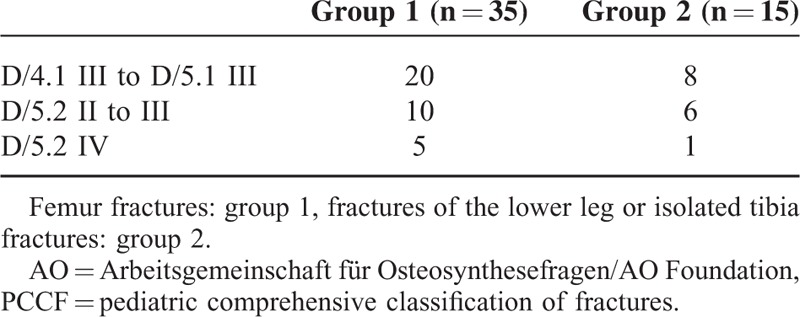
Fracture Patterns Based on the AO Pediatric Comprehensive Classification of Long Bone Fractures (PCCF)^13^

### Type of Injury

There was a single open femur fracture and 6 open lower leg or tibia fractures. Based on the classification of open fractures according to Gustilo et al,^14^ five open fractures in group 2 were of grade I and 1 was of grade II. The open fracture in group 1 was grade I. In group 1 and group 2, 4 and 3 of these children incurred associated injuries, respectively (Table 3). All children with associated injuries, except 1 in group 1, were initially not allowed to bear weight. All children with associated injuries in group 2 were additionally immobilized using a plaster cast. However, in group 2, 2 of 3 children achieved partial weight bearing at around the same time as the children without associated injuries. Time until full weight bearing was only documented in 2 of 3 children. One of these children achieved full weight bearing in <8 weeks (7.7 weeks). In group 1, 2 children achieved first partial weight bearing in the first 2 weeks after the operation, whereas 2 children were able to tolerate full weight bearing within 5 weeks.

**TABLE 3:**
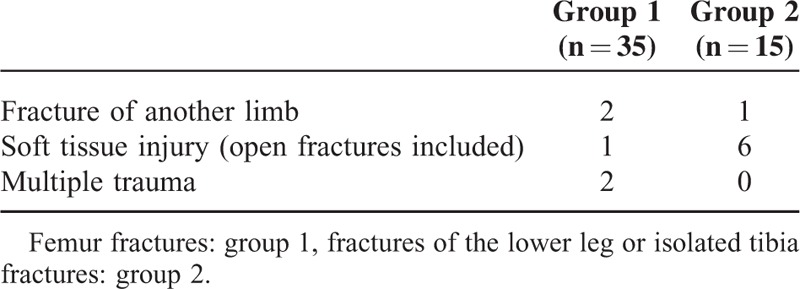
Associated Injuries

### Postoperative Complications

**FIGURE 1:**
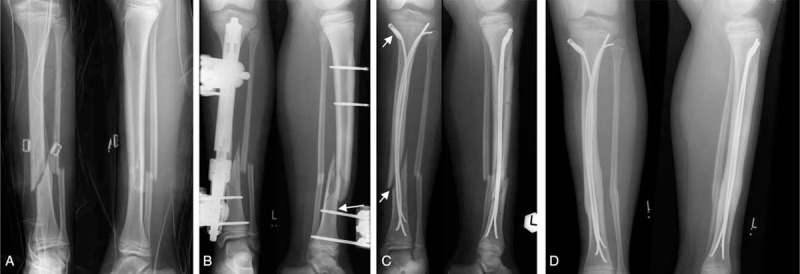
An 11-year-old girl incurred a first-degree open long oblique fracture of the left lower leg in a pedestrian car collision (A). Due to multiple injuries and the 1° open fracture of the lower leg, she was initially treated with an external fixator (B). Stabilization with ESIN was carried out 6 days after the injury. After closed reduction, 3 nails were inserted together with 1 EndCap to increase the stability of the osteosynthesis (C). Five degrees of antecurvation and a valgus angulation of 8° were accepted in this child. Five days postoperatively, the child slipped and fell, provoking minimal displacement of a nail. Consequently, a plaster cast was applied, and further mobilization was uneventful. Radiological consolidation was noted after 6 months (D). The valgus angulation and antecurvation had disappeared, and there was no shortening at the fracture site. ESIN = elastic stable intramedullary nails/nailing.

Postoperative complications occurred in 3 of 15 children (20%) who had sustained lower leg fractures. Two children developed symptoms of a beginning compartment syndrome, and 1 of these children suffered an acute lower leg compartment syndrome which was managed surgically. Another child sustained a fall during the early postoperative physical rehabilitation program, resulting in minimal displacement of the implants (Figure 1). None of the children who had suffered a femur fracture experienced an early postoperative complication.

### Length of Hospital Stay

Median length of hospital stay was 5 days in group 1 (range: 1–57 days) and 7 days in group 2 (range: 1–46 days).

### Postoperative Plaster Cast Immobilization

In children of group 2 treated without postoperative plaster cast immobilization, we observed partial and full weight bearing after a median of 1.3 weeks (range: 0.1–5.1 weeks) and 7.4 weeks (range: 5.3–21.7 weeks), respectively.

### Postoperative Time Interval Until Partial and Full Weight Bearing

**FIGURE 2:**
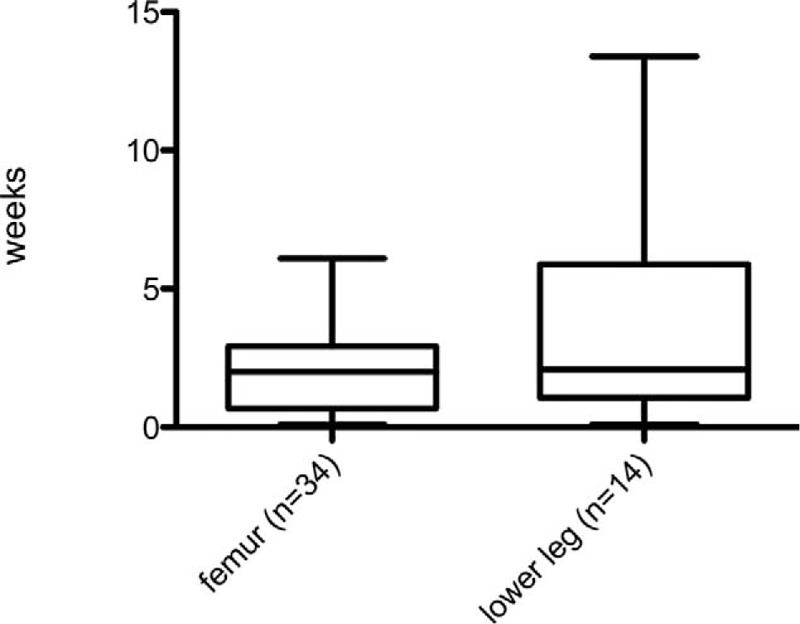
Time until initiation of partial weight bearing was documented in 48 children. First partial weight bearing was recorded after a median duration of 2 weeks in the group of children who had sustained femur fractures and lower leg fractures.

**FIGURE 3:**
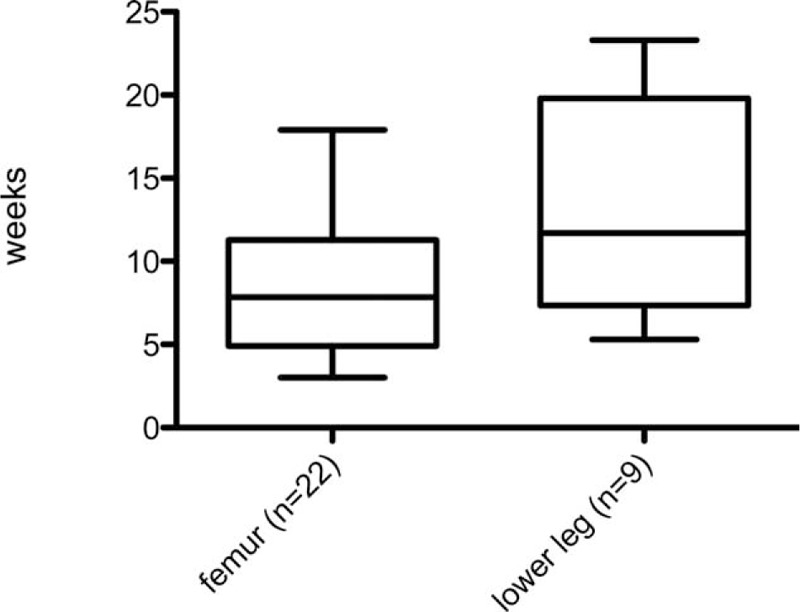
Time until full weight bearing (n = 31 children). Time until full weight bearing was 7.8 weeks in children who had suffered a fracture of the femur and 11.7 weeks in children who had suffered fractures of the lower leg or tibia.

Figures 2 and 3 show the postoperative time until partial and full weight bearing, respectively. All children included in this study achieved full weight bearing. The postoperative time interval until initiation of partial weight bearing with transverse and short oblique femur fractures (median: 2.4 weeks; range: 0.1–6.3 weeks) did not differ significantly (U = 0.34; n.s.) from that with long oblique, spiral, and multifragmental femur fractures (median: 2.2 weeks; range: 0.1–9.4 weeks). Similarly, the time until partial weight bearing with transverse and short oblique lower leg and tibia fractures (median: 1.7 weeks; range: 0.1–13.4 weeks) was not significantly shorter than that with more complex lower leg fractures (median 4.1 weeks; range 1.3–9.1 weeks) (U = 1.34; n.s.)

However, comparison of the postoperative time interval until full weight bearing with transverse and short oblique femur fractures (median: 4.4 weeks; range: 0.1–9.1 weeks) to that with long oblique, spiral, and multifragmental femur fractures (median: 6.8 weeks; range: 2.9–13.9 weeks) showed a significant difference (*U* = 1.83; *P* = 0.04). Similarly, transverse and short oblique lower leg and tibia fractures required significantly less time until full weight bearing (median: 4.1 weeks; range 2.7–6.0 weeks) than complex lower leg fractures (median: 6.1 weeks; range: 1.3–12.9 weeks; *U* = 1.79; *P* = 0.04).

**FIGURE 4:**
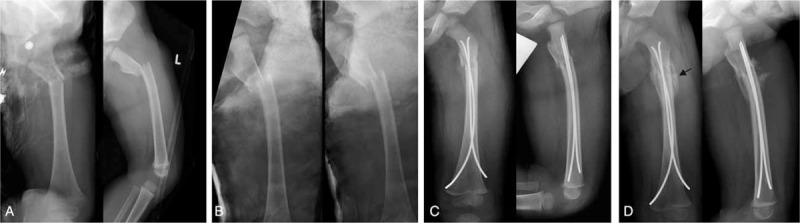
A 3-year-old boy sustained a subtrochanteric transverse femoral fracture. (A) Spiral fracture of the distal tibial shaft and buckle fracture of the distal fibula shaft after having been run over by a motor vehicle. Primarily, all fractures were treated nonoperatively with a hip spica cast. After 3 days of hip spica cast treatment, the angulation at the fracture site was not corrected (B), and the decision was made to reduce and stabilize the femur fracture using the ESIN technique (C). Radiological union of the femur fracture was documented 1 month postoperatively. We noted massive callus formation bridging at the fracture site (D). ESIN = elastic stable intramedullary nails/nailing.

Two boys in group 1 (aged 6.8 years and 3.2 years) achieved early full weight bearing (after 3.0 and 3.3 weeks, respectively), defined as postoperative time of <6 weeks until full weight bearing. In the younger boy, a lateral EndCap (Synthes–De Puy, West Chester, PA) was inserted which provided sufficient anchoring of the nail end in the cortical bone. The older boy had sustained a spiral fracture with a shortening of ∼8 mm with a long contact area of the fracture line, resulting in massive callus formation and early fracture consolidation. The younger boy, who had sustained a subtrochanteric transverse fracture, showed massive callus formation in the postoperative radiographs (Figure 4).

Additionally, we recorded early full weight bearing (after 5.3 weeks) in 1 boy aged 6.5 years in group 2 who had sustained a short oblique fracture (D/4.1 III) of the lower leg without associated injuries. In the postoperative biplanar radiographs, we observed a well-reduced fracture with shortening of the fibula of ∼3 mm and minimal valgus malalignment of the tibia (6°).

Delayed full weight bearing, characterized by full weight bearing occurring >8 weeks after the injury, was noted in 10 of 35 children (7 girls; 3 boys) of group 1. Median age of these children was 8.1 years (range 3.6–15.7 years). One of these children had sustained multiple trauma, and another child had suffered an additional fracture of the radius. In 9 of these children, a closed reduction had been performed. One lower leg fracture was initially stabilized by application of an external fixator. No child of group 1 was immobilized after surgery using a plaster cast, and no complications occurred during postoperative rehabilitation. One child required a reoperation with osteotomy to correct an external rotational malalignment of 21° at the fracture site. The preoperative radiographs of these children revealed a comminuted fracture (D/5.2 IV) in 2 children and a spiral fracture in 2 children (D/5.2 III), indicative of a more complex type of fracture.

**FIGURE 5:**
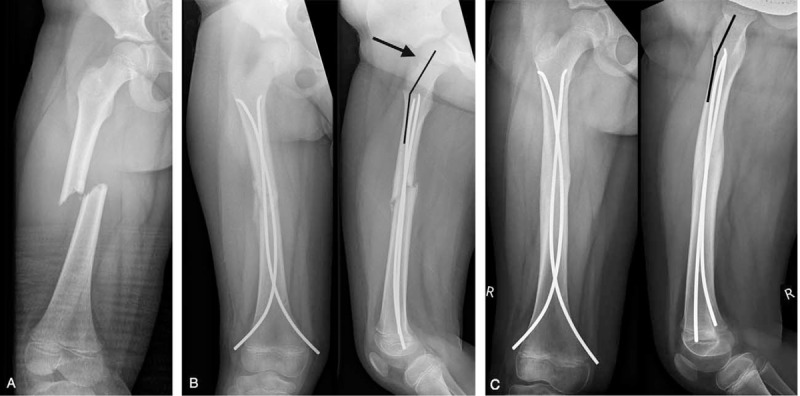
A 5-year-old girl sustained a short oblique fracture of the femur when injured in a pedestrian-motor vehicle collision. (A) The radiographs obtained 4 weeks after the injury showed moderate retrotorsion of the femoral neck. This external rotational malalignment should not have been accepted (B). Union of the fracture was confirmed by radiographs after 5 months, and there was minimal retrotorsion of the femoral neck (indicated by the angle) in the lateral view indicative of minimal external rotational malalignment (C).

**FIGURE 6:**
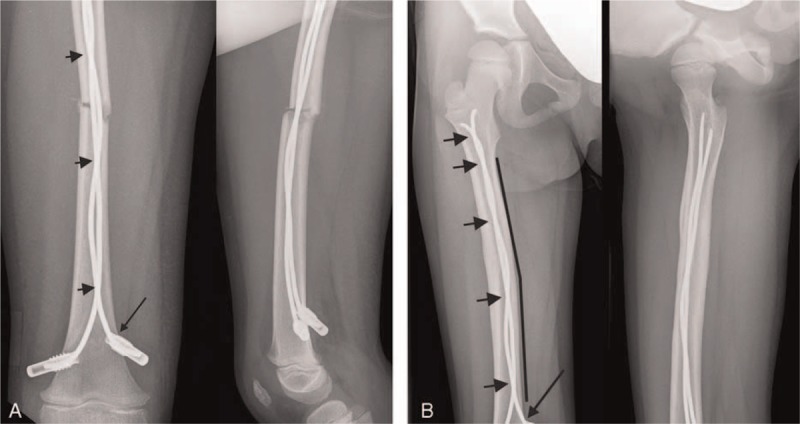
A 5-year-old girl suffered a transverse fracture of the femur during a fall down the stairs. Elastic intramedullary stabilization was performed on the day of injury. The radiograph obtained 10 days later showed a twisting of the nails (“corkscrew” phenomenon). Two EndCaps (long arrow) were inserted to increase the biomechanical stability of the osteosynthesis (A). Radiological consolidation of the fracture was documented after 5 months. There was a residual valgus angulation of 8°. The multiple crossings of the nails are indicated by arrows (B).

In 4 patients of group 1, postoperative retrotorsion of the femoral neck occurred (Figure 5). One adolescent had closed physes at the time of intervention, and consolidation of the fracture was partially delayed. In 4 children, the protruding distal nail ends were left too long and subsequently interfered with the knee retinaculum. In 1 child, we noted a twisting of the 2 nails (“cork screw phenomenon”; Figure 6). In another child, we found a postoperative dehiscence of ∼6 mm at the fracture site causing delayed callus formation and consolidation of the fracture. In all of these children, nails of adequate diameters had been used.^15^ In 2 children, poor premoulding of the nails was noted. In 4 children, TEN (Titanium Elastic Nail) EndCaps (Synthes GmbH, Oberdorf, Switzerland) were applied to anchor the distal ends of the nails and to prevent the backing out of the nail ends. We observed migration of 1 EndCap in 1 child, and in 2 children we noted poor anchoring of the EndCaps in the metaphyseal bone. In 3 children, a very narrow intramedullary cavity was present.

Two girls and 3 boys of group 2 did not regain full weight bearing capacity within 8 weeks after the injury. Median age of these children was 9.4 years (range 6.2–11.1 years). One of them had sustained an isolated fracture of the tibia, and 2 had suffered open fractures (grade I and grade II). One of these children also suffered an avulsion of the skin of the foot and lower leg. One child was initially treated with an external fixator for 6 days (Figure 1).

Four children were immobilized using a cast after the operation. Duration of immobilization was between 2 days and 4 weeks. In the early postoperative phase, 2 children in this group experienced complications; 1 sustained an acute compartment syndrome and the other suffered a fall with a consecutive painful minimal displacement of 1 implant. In 1 child, postoperative external malrotation of the tibia of 10°, which was confirmed by computed tomography, necessitated a corrective osteotomy. In the radiological documentation of these children, we noted that all fractures were comminuted or severely displaced (D/5.2 III to IV). In 1 child, the intact fibula hindered timely consolidation of the fracture of the tibia. In 2 children, we had used EndCaps and in 1 child we had used 3 nails in an attempt to increase the stability of the osteosynthesis. Postoperatively, we noted a valgus deformity of 5 and 10° in 2 children that was remodeled during the course of progressive fracture healing.

### Resumption of School Sports

All children, except 5 children who were not required to attend school at the time of the fracture, regained the capacity for participating in school sports; however, in 13 children the time of resumption of school sports was not well documented. In group 1 and group 2, 22 patients and 6 patients, respectively, were followed after the removal of the implants and underwent final clinical examination. Resumption of school sports was seen after a median of 13.8 weeks (range 6.0 to 39.1 weeks) in group 1 and 17.8 weeks (range 8.0 to 31.1 weeks) in group 2. Four of 7 children of group 2 immobilized in a cast were followed up. One of these children complained about pain at sports at follow-up.

### Radiological Consolidation

Complete radiological consolidation was noted after a median duration of 19.0 weeks (range 7.1–58.9 weeks) in group 1 and after 17.3 weeks (range 7.6–33.6 weeks) in group 2. In 5 children, removal of the implants took place elsewhere.

### Implant Removal

In femur fractures, the nails were removed after a median duration of 5.9 months (range 2.1–16.9 months) and in lower leg or isolated tibia fractures after a median of 7.2 months (range 2.2–10.5 months). Because many children were followed up by their pediatrician, we were not able to report complete data on the functional outcome in all children.

### Follow-up Results

Table 4 summarizes the subjective complaints and objective findings at follow-up examinations, which were performed together with plain x-rays before planning implant removal.

**TABLE 4:**
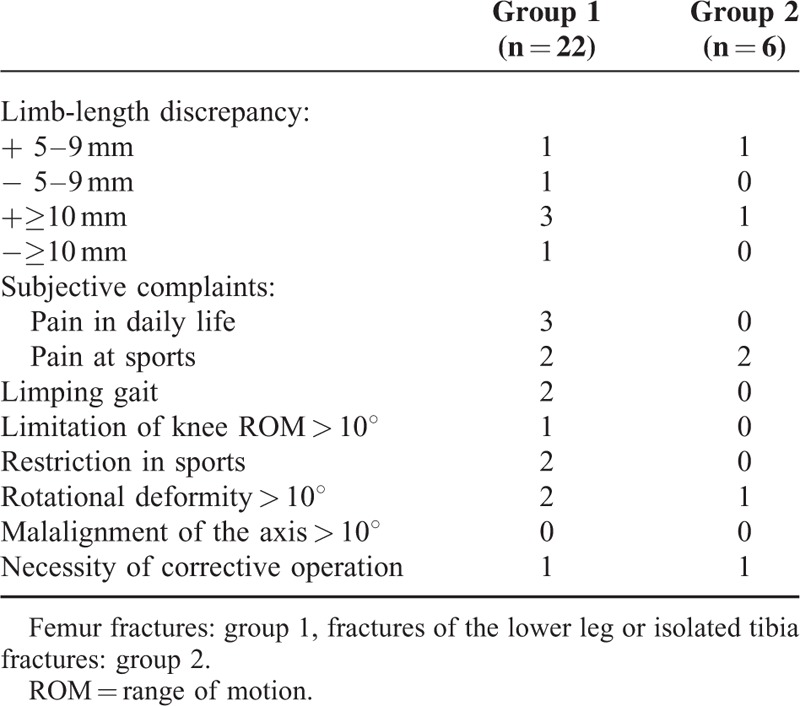
Functional Outcome After a Median Time Span of 8.3 Months (Range: 2.3–74.5 Months)

## DISCUSSION

Initially, the invention of ESIN gave reason to hope that children who sustained a displaced long-bone shaft fracture would benefit from shortened hospitalization and rapid functional rehabilitation. The biomechanical idea of transforming bending forces by intramedullary nails into compressing and distracting forces and thus stimulating callus formation together with limiting shearing movements at the fracture site promised high effectiveness and early mobilization.^5,11^

In our study, median duration of hospitalization was 5 days in femur fractures and 7 days in lower leg and tibia fractures, which compares well to the results in the literature.^7,16–18^ In a study in 91 children with femoral fractures, Ho et al^16^ reported that during the immediate postoperative phase, 57% of patients were discouraged from weight bearing, 19% were allowed partial weight bearing, 24% were allowed weight bearing as tolerated, and 1 patient was allowed full weight bearing. These recommendations are similar to our postoperative treatment regimen (Table 1). In accordance with Narayanan et al^19^ who recommended to use ESIN with additional external immobilization in complex fractures, we postoperatively applied a plaster cast in 7 of 15 children who had suffered lower leg or tibia fractures. In line with the suggestion of Vallamshetla et al, who applied a plaster cast for postoperative immobilization in all children treated with ESIN, we started weight bearing after plaster cast removal at a median of 2 weeks after the operation.^20^ The indications making additional immobilization necessary were not detailed in the study of Vallamshetla et al. In our review of the radiographs, we hypothesized that a fracture location in the distal third of the tibia, or the presence of a third fragment, indicative of higher postoperative instability may be considered eminent reasons to apply a plaster cast in the early postoperative period. This hypothesis needs to be confirmed in a larger study population.

Previous studies indicated that ESIN bears limitations in the treatment of complex fracture patterns.^1,21^ Maier and Marzi described the start of weight bearing after ESIN stabilization of femur fractures in children in the first days after the intervention.^22^ However, these authors stressed that more complex fractures of the femoral shaft required a prolonged period of time until full weight bearing was achieved.^22^ Thus, these patients do not benefit from the biomechanical advantages of ESIN in the very early postoperative stages.

Flynn et al^23^ compared the outcome of children with a femoral fracture treated with ESIN to the outcome of femur fractures managed with traction and spica cast treatment. With a mean age of 10.2 years (range 6–16 years) in the ESIN group, these children were slightly older than the children in our cohort. Flynn et al reported that in their group, the children were able to walk with aids and bear partial weight after 2 weeks,^23^ which is similar to our findings. In the study by Bukvić et al, who examined the validity of ESIN in long-bone fractures in children and adolescents, 28 children with a fracture of the lower leg began with partial weight bearing after 6.6 days in the mean.^24^ This period is much shorter than that observed in our patients, but Bukvić et al only included children with transverse or oblique fractures.^24^

The time until full weight bearing was not documented for all children in our retrospective study due to incomplete hospital records and subsequent follow-up by resident pediatricians and practitioners. The available data showed that full weight bearing was achieved after a median duration of 7.8 weeks (range 3.0–17.9 weeks) in group 1 and after 11.7 weeks (range 5.3–23.3 weeks) in group 2. We are unable to estimate the influence of missing follow-up data on the results obtained. Compared to the only prospective study investigating ESIN in pediatric femoral fractures,^18^ which reported a mean time until full weight bearing of 7 weeks (range 3–10 weeks), the children in our study required slightly more time to achieve full weight bearing.

We compared our results of group 2 to data reported by Vallamshetla et al, Gicquel et al, and Kubiak et al,^2,20,25^ who investigated tibial fracture stabilization using ESIN. Gicquel et al reported that full weight bearing was achieved after a mean duration of 6 weeks.^2,20,25^ In this study, 45 children had been treated with ESIN. Transverse fractures were seen most frequently (23 children), but the distribution pattern of the fractures in these children was not described in detail. Furthermore, Gicquel et al did not report any postoperative recommendations for weight bearing.^2^ Therefore, a comparison to our results was not possible. On the other hand, Vallamshetla et al placed all patients in a plaster cast postoperatively and suggested a nonweight bearing period of 4 to 6 weeks.^2,20,25^ Consequently, full weight bearing was reported about 6 weeks after cast removal. Kubiak et al allowed partial weight bearing with crutches immediately after the operation if there was an overlap of the fragments of >50%.^2,20,25^ However, the time until full weight bearing was not reported; instead the authors documented the duration until fracture union.

Partial weight bearing in children who had sustained lower leg fractures and were immobilized in a plaster cast was observed 1 month after the onset of partial weight bearing in children treated without plaster cast. A similar time difference (+4.8 weeks) was noted between these groups regarding the onset of full weight bearing. The time interval until partial weight bearing in the children of group 2 not treated with cast immobilization was shorter when compared to results reported by other authors.^20,24,25^ However, the children of group 2 not treated with postoperative cast immobilization had sustained fractures type D/4.1 III and D/5.1 III similar to the distribution of fracture types reported by Bukvić et al who reported an onset of weight bearing after 6.6 days.^13,24^ In accordance with Dietz and Schlickewei and Bopst et al, we noted reduced efficacy of ESIN to facilitate early full weight bearing in more complex long bone shaft fractures of the lower extremities.^1,21^ Due to the limited number of patients, these data must be interpreted with caution.

The indications for postoperative plaster cast immobilization in children who underwent ESIN stabilization of fractures of the lower leg are not clearly described in the literature. Vallamshetla et al and O’Brien et al placed all children after ESIN of the tibia in a plaster cast for several weeks and allowed “protected” weight bearing in the walking plaster when a callus formation was seen and the fracture site did no longer hurt.^20,26^ O’ Brien et al justified the cast application on the grounds of the patient's comfort and protection.^26^ However, Ligier et al did not recommend the application of a cast except for the postoperative correction of an axial deviation.^11^

In total, 3 of our 50 children experienced early full weight bearing within 6 weeks after the operation. Children with short oblique or transverse fractures may benefit from early weight bearing since complicated fracture patterns often require immobilization in the early postoperative period.^1,21^

Ligier et al and Maier et al recommended the beginning of partial weight bearing and subsequent full weight bearing after 3 weeks or 4 to 6 weeks, respectively.^4,5^ Consequently, delayed full weight bearing was characterized in the present study by onset of full weight bearing after >8 weeks. In the present study, 10 children in group 1 and 5 children in group 2 met this criterion. The 10 children in group 1 sustained rather complex fractures and in 5 children, a problem involving protruding distal nail ends had occurred, a complication which was previously described by Slongo.^27^ The nail ends protruded into the region of the iliotibial tract and the retinaculum of the patella with a consecutive painful discomfort limiting the mobility of the knee. Furthermore, slight nail migration during rehabilitation was observed in 1 child (2%) who had suffered a femur fracture and in whom nails without EndCaps were used. Retrospectively, we concluded that some of these children might have benefited from EndCaps^28^ or similar nail end stabilization devices (e.g. HSN-esin-Hofer sliding Nail, Hofer GmbH & CoKG, Fürstenfeld, Austria) designed for stabilization of complex long-bone shaft fractures in children. In 1 child who had sustained a femur fracture, we noted corkscrewing of the nails. This phenomenon, also described by Slongo in a study examining common errors in ESIN treatment, impedes the biomechanical function and stability of the nails.^27^ By twisting of the nails, they act biomechanically as 1 single nail with subsequent reduction of stability.

Four of 35 children (11.4%) suffered associated injuries in combination with a femur fracture. Therefore, due to the small number of children who sustained accompanying injuries, the influence of accompanying injuries on the onset of partial or full weight bearing could not be assessed.

All children who experienced delayed full weight bearing after a fracture of the lower leg had been immobilized in a plaster cast for up to 4 weeks postoperatively because of their complex fracture patterns. One of these children also suffered a complex fracture of the foot and a decollement injury, which precluded early mobilization. In our study, complete consolidation documented radiologically occurred after a median duration of 19.0 weeks in group 1 and 17.3 weeks in group 2. This is notably longer than the durations reported in the literature.^16,25,29,30^ In a literature review, Baldwin et al^10^ reported a wide range of time spans until consolidation of the fracture; they included 16 studies examining the treatment of femoral fractures in school children and found the consolidation time to range from 4 to 11 weeks.

Although we reported slower consolidation, the time interval between insertion and removal of the implant was similar to the findings in the literature. Bar-On et al^18^ removed the implants after an average of 7 months in children who had suffered a femoral fracture. Vallamshetla et al^20^ reported that nails were removed after 6 to 9 months in children who incurred a fracture of the lower leg.

Only 1 child was allowed to bear weight immediately after the operation. Physiotherapy was applied in all children with similar intensity and frequency. In hospital, physiotherapy was offered once a day and in the outpatient setting 1 h to 2 h per week. Because all children underwent physiotherapy, we are unable to assess the effect of postoperative physiotherapy compared to spontaneous mobilization.

Functional outcome was documented for 28 children in our study. Because we did not apply a specific questionnaire, for example, the Paediatric Outcomes Data Collection Instrument (PODCI)^31,32^ during follow-up to assess functional outcome, pertinent comparison with other studies concentrating on the satisfaction of patients after ESIN was not possible. Limb-length discrepancies, which were measured during the regular clinical follow-up examinations in our study, ranged from 7 mm to 15 mm (Table 4), which is acceptable considering the guidelines developed by Kasser and Beaty applied in the study by Flynn et al.^23^ In follow-up examinations, we identified 2 children with rotational malalignment. These children subsequently underwent a corrective osteotomy.

The results of this study should be generalized with caution. Our investigation represents a single-centre study in a retrospective setting, and the postoperative treatment regimen and follow-up examinations were not based on a precisely defined protocol. The number of children documented until full weight bearing dropped from 50 to 31. Both the intra- and inter-group heterogeneities were marked, thus making the power of comparison low. Nevertheless, our study suggests that ESIN is a safe method to treat unstable, displaced long-bone shaft fractures of the lower extremities in children. With increasing complexity of the fracture pattern, the expected early functional rehabilitation after ESIN treatment of long-bone shaft fractures of the lower extremities seems to forfeit the effectiveness, and full weight bearing seems to be delayed. We recommend performing prospective studies to confirm our findings.
